# Global parameter search reveals design principles of the mammalian circadian clock

**DOI:** 10.1186/1752-0509-2-22

**Published:** 2008-02-29

**Authors:** James CW Locke, Pål O Westermark, Achim Kramer, Hanspeter Herzel

**Affiliations:** 1 Institute for Theoretical Biology, Humboldt-University Berlin, 10115 Berlin, Germany; 2 Department of Systems Biology, University of Warwick, Coventry, CV4 7AL, UK; 3 Laboratory of Chronobiology, Charité Universitätsmedizin Berlin, 10115 Berlin, Germany; 4 Division of Biology and Department of Applied Physics, California Institute of Technology, Pasadena, California 91125, USA

## Abstract

**Background:**

Virtually all living organisms have evolved a circadian (~24 hour) clock that controls physiological and behavioural processes with exquisite precision throughout the day/night cycle. The suprachiasmatic nucleus (SCN), which generates these ~24 h rhythms in mammals, consists of several thousand neurons. Each neuron contains a gene-regulatory network generating molecular oscillations, and the individual neuron oscillations are synchronised by intercellular coupling, presumably via neurotransmitters. Although this basic mechanism is currently accepted and has been recapitulated in mathematical models, several fundamental questions about the design principles of the SCN remain little understood. For example, a remarkable property of the SCN is that the phase of the SCN rhythm resets rapidly after a 'jet lag' type experiment, *i.e*. when the light/dark (LD) cycle is abruptly advanced or delayed by several hours.

**Results:**

Here, we describe an extensive parameter optimization of a previously constructed simplified model of the SCN in order to further understand its design principles. By examining the top 50 solutions from the parameter optimization, we show that the neurotransmitters' role in generating the molecular circadian rhythms is extremely important. In addition, we show that when a neurotransmitter drives the rhythm of a system of coupled damped oscillators, it exhibits very robust synchronization and is much more easily entrained to light/dark cycles. We were also able to recreate in our simulations the fast rhythm resetting seen after a 'jet lag' type experiment.

**Conclusion:**

Our work shows that a careful exploration of parameter space for even an extremely simplified model of the mammalian clock can reveal unexpected behaviours and non-trivial predictions. Our results suggest that the neurotransmitter feedback loop plays a crucial role in the robustness and phase resetting properties of the mammalian clock, even at the single neuron level.

## Background

Almost all organisms have evolved to co-ordinate their activities with the many changes in the environment caused by the earth's rotation [1]. A circadian clock generates biological rhythms with a period of approximately 24 hours (h), and is found in most eukaryotes and photosynthetic bacteria. In mammals, a central pacemaker exists in the suprachiasmatic nucleus (SCN) of the hypothalamus, which generates and communicates a circadian rhythm to other parts of the brain, and to peripheral tissues. The clock is entrained to the day/night cycle, with the SCN receiving light information from the retina through the retinohypothalamic tract, but is also self-sustained under constant conditions [2].

The SCN consists of two nuclei located above the optic chiasm, each containing around 10000 neurons. Dissociated neurons are able to produce circadian oscillations under constant conditions, although less robustly, with a period of between 20 and 28 h [3,4]. The oscillation in individual neurons is generated by a network of interlocking feedback loops involving clock genes and proteins, similar to that seen in other organisms. Key components in this machinery are the clock proteins PERIOD1 (PER1), PER2 and CRYPTOCHROME1 (CRY1), CRY2, and the transcription factors CLOCK and BMAL1. These feedback loops have been extensively studied [2] and modelled [5-7]. A coupling mechanism must exist between the individual neurons in order to generate a robust 24 h collective self-sustained rhythm under constant conditions of the SCN tissue. Several candidate coupling factors have been discussed [8]. Recently, vasoactive intestinal polypeptide (VIP) has been implicated as one of the possible coupling factors, since mutations in genes coding for VIP as well as for its receptor VPAC2 disrupt normal synchronisation of the clock neurons within the SCN, and maintenance of molecular timekeeping within individual SCN neurons [9]. It has also recently been shown that SCN intercellular coupling can make the system more robust against genetic perturbations [10].

Populations of individual oscillators with variable periods have been shown to be synchronised by a coupling factor in a variety of contexts [11], including pacemaker cells in the heart, synchronization of firefly flashes, and circadian rhythms in *Drosophila*. The properties of coupled oscillators have been researched in great detail [12,13], and have long been studied in relation to circadian rhythms [14]. Several theoretical studies have also been made specifically examining the coupling mechanism of the SCN [15-19]. Gonze et al. previously studied theoretically the synchronising properties of the SCN, and showed that a global coupling mechanism relying on a mean field of neurotransmitter is sufficient to synchronize a population of 10,000 cells [17]. These results have been generalized to more complex single cell models and a variety of coupling schemes [18]. A recent theoretical study has also examined in detail the role of VIP in synchronising the SCN [19].

A fundamental property of the SCN is that the phase of the SCN rhythm resets rapidly after a 'jet lag' type experiment, where the light/dark (LD) cycle is abruptly advanced or delayed by several hours. It has been shown experimentally that the levels of the clock protein PER1 in the SCN are almost completely re-entrained within one day of a 6 h delay or advance in LD cycle, being completely reset in around 6 days [20-22]. It remains an open question how ~10000 neurons are able to respond so quickly to a shift in the entraining signal, even though phase response curve experiments have shown that the SCN rhythm appears to be phase shifted only by a maximum of a few hours by light and neurotransmitter pulses. A Phase Response Curve (PRC) is a plot of phase-shifts as a function of circadian phase of a stimulus such as light pulses, temperature pulses, or pulses of drugs or chemicals. The SCN shows a weak (type 1) PRC to pulses of light and a variety of neurotransmitters including VIP and gastrin-releasing peptide (GRP) [23-27].

The phenomenon of fast phase resetting, and in fact, the balance between the two primary synchronisers of the SCN, light, and intrinsic neurotransmitters, is not well understood. In the Gonze et al. model, entrainment to a 12:12 h LD cycle resulted in quasiperiodic behaviour, with small variations in the amplitude from cycle to cycle. A shift in LD cycles caused very long transients, taking around twenty days for the phase to reset to a 6 h shift in LD cycle, rather than the 1–6 days seen in experiment (see Additional file 1: Supplementary Figure S1, Additional file 2: Supplementary figure legends). However, it remains unclear whether this behaviour is a general feature of the network equations (described in Additional file 3, which contains the supplementary information), or if this is due to the parameters chosen.

In this work, we use a global parameter search scheme [28,29] to investigate the resetting properties of a simplified model of the SCN [17]. We investigate the top 50 parameter sets found using our parameter optimization scheme, and show that the neurotransmitter feedback loop can be crucial for oscillations, even at the single cell level. We also show that if the neurotransmitter drives a system of damped, rather than self-sustained oscillators, it is possible to achieve fast phase resetting in simulated 'jet lag' type experiments even with this simple model of the SCN. Finally, we go on to show that fast resetting properties are still possible even in the more experimentally realistic case of only a sub fraction of the oscillators being light-activated.

## Results and Discussion

### Global parameter screen reveals that the neurotransmitter feedback loop can be essential for single neuron oscillations in the SCN

**Figure 1:**
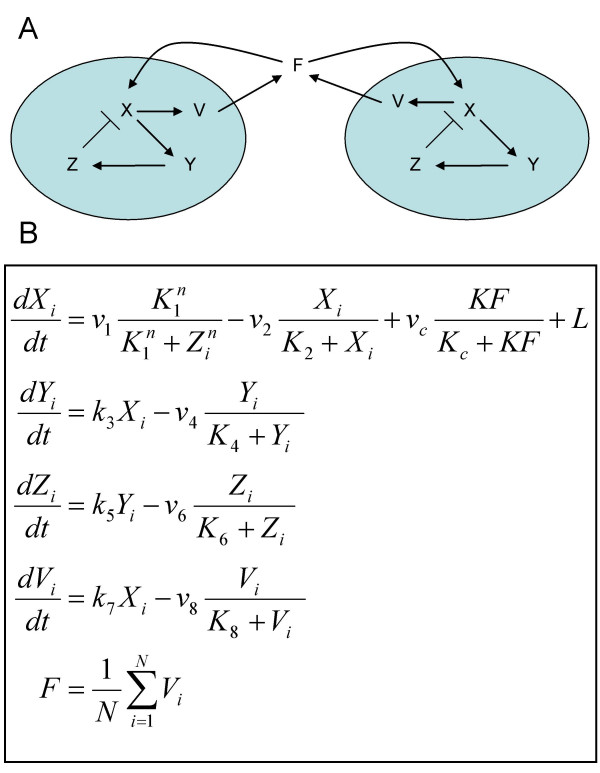
**The synchronized oscillator model**. A. Network diagram showing two representative coupled clock cells. Within each clock cell, a clock gene mRNA (*X*) produces a clock protein (*Y*) which, in turn, activates a transcriptional inhibitor (*Z*), closing a negative feedback loop. Clock cells synthesize a neuropeptide denoted by *V*, induced by the activation of the clock gene (*X*). The mean field levels of the neuropeptide (*F*) then acts as a synchronising factor by activating *X *transcription both in an autocrine and paracrine manner. B. Network equations for a system of N oscillators (denoted by (i = 1, 2, ..., N) as described in Figure 1A. Light (*L*) acts on the system by activating *X *transcription. For detailed description, see [17], and Additional file S3.

We carried out a global parameter screen of a previously developed simplified model of the SCN [17] in order to study in depth the design principles of the mammalian circadian clock. In this model (Figure 1A), within each clock cell, a clock gene mRNA (*X*) produces a clock protein (*Y*) which goes onto activate a transcriptional repressor (*Z*). *Z *represses the transcription of the clock gene *X*, closing a negative feedback loop. It is assumed that clock cells synthesize a neuropeptide denoted by *V *and that production is induced by the activation of the clock gene (*X*). The neurotransmitter release is assumed to be fast in comparison to the timescale of the oscillations (~24 h). The neurotransmitter levels thus become homogeneous, resulting in an average neurotransmitter level, or a mean field *F*. The mean field level of the neuropeptide then acts as a synchronising factor by activating *X *transcription both in an autocrine and paracrine manner (see Figure 1B for model equations, and Additional file 3 for detailed description). In reality, the SCN is much more complex than this simplified model. The clock circuit within each cell contains multiple feedback loops, and the strength of the neurotransmitter feedback appears to vary from neuron to neuron. For example, only a subsection of the actual SCN neurons express the VPAC2 receptor for the neurotransmitter VIP [30]. However, by analysing in depth an idealized model such as this, it is often possible to gain important insights about putative mechanisms [31].

In order to estimate the 16 unknown parameters in this model, we defined a cost function (CF) that gave a low score for solutions that had a limit cycle oscillation of the mean field in constant conditions with a period of approximately 24 h, and were well synchronised (see Additional file 3, and [29] for details of the optimisation scheme). We calculated the CF for a million candidate parameter sets chosen quasi-randomly, and then we picked the parameter set giving the lowest CF score as our optimal solution for further detailed analysis. We also used the top 50 parameter sets to further explore the generalised properties of synchronisation.

**Figure 2:**
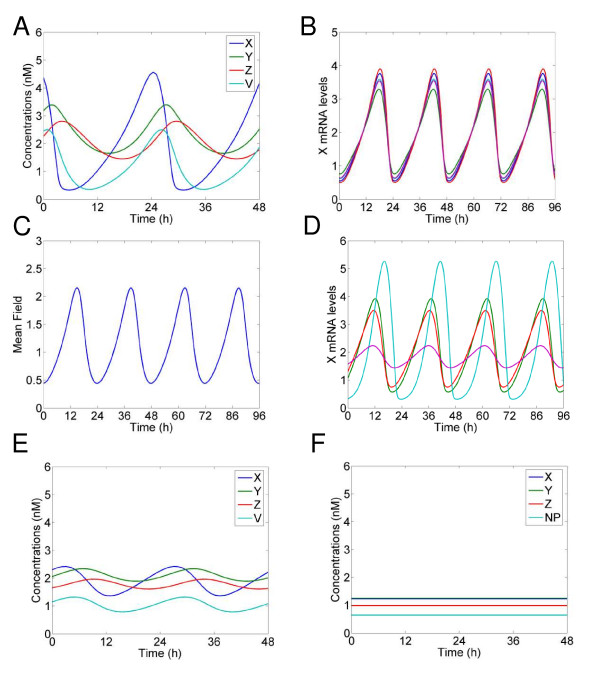
**Neurotransmitter can drive oscillations at the single neuron level**. A. Simulation of one circadian oscillator using optimal parameter set *v*_1 _= 6.8355 nM/h; *K*_1 _= 2.7266 nM; *n *= 5.6645; *v*_2 _= 8.4297 nM/h; *K*_2 _= 0.2910 nM; *k*_3 _= 0.1177/h; *v*_4 _= 1.0841 nM/h; *K*_4 _= 8.1343 nM; *k*_5 _= 0.3352/h; *v*_6 _= 4.6645 nM/h; *K*_6 _= 9.9849 nM; *k*_7 _= 0.2282/h; *v*_8 _= 3.5216 nM/h; *K*_8 _= 7.4519 nM; *v*_c _= 6.7924 nM/h; *K*_c _= 4.8283 nM; *K *= 1; and *L *= 0. Concentrations are expressed in nM. B. Simulation of 200 circadian oscillators using the optimal parameter set. Oscillations of *X*_i _(in nM) for 5 randomly chosen oscillators. Different individual periods were obtained by rescaling rate constants by a scaling factor *τ*_i_, *i *= 1, ..., *N*. The values of *τ*_i _are drawn randomly from a normal distribution of mean 1.0 and standard deviation 0.05. C. Oscillation of the mean field of the neurotransmitter (*F*). D. Simulation of 200 circadian oscillators with highly variable periods using optimal parameter set. The simulation was carried out as in B but with a standard deviation of 0.25 for the periods. E. Simulation of one circadian oscillator using the optimal parameter set. Parameters same as in 1A) except coupling strength constant, K, reduced to 0.8. F. Simulation of one circadian oscillator using the optimal parameter set. Parameters same as in 1A) except coupling strength constant, K, reduced to 0.

Figure 2A shows a simulation using our optimal parameter set of *X*, *Y *and *Z *levels, as well as neurotransmitter levels, for a single neuron under constant dark conditions. *X *levels peak 3–4 h before *Y*, and there is another 2–3 h delay between *Y *levels and *Z *similar to experimental findings. The neurotransmitter has a similar profile as *X*, but is slightly delayed. Figure 2B shows *X *levels for 5 randomly selected oscillators from a set of 200 coupled oscillators. Each individual oscillator has its parameters rescaled by a scaling factor to represent the fluctuations in period and amplitude seen experimentally in isolated neurons [3]. The oscillators are extremely well synchronised, with the mean field of the transmitter oscillating with a period of 24 h (Figure 2C). The oscillators remain synchronised even when the individual parameters are rescaled to represent 5 fold larger fluctuations in period (Figure 2D). A simulation of 10000 coupled oscillators similar to the total number of neurons in the SCN was also extremely well synchronised (data not shown), but for this study we concentrate on a more numerically tractable number of 200 oscillators for in depth analysis.

Simulations using our optimal parameter set initially appear similar to simulations using handpicked parameters [17]. However, in contrast to that work, a reduction of neurotransmitter strength leads to a very rapid reduction in the amplitude of the oscillation, even for a single neuron simulation. A reduction in neurotransmitter strength could be caused experimentally by several factors, such as pharmacological or genetic perturbations of the VIP/VPAC2 signalling pathway. If the coupling constant is reduced to 80% of the original value a single neuron generates a self-sustaining oscillation, but with a much smaller amplitude (Figure 2E), and complete removal of the neurotransmitter loop results in arrhythmia (Figure 2F). This means the neurotransmitter positive feedback loop substantially contributes to even single cell oscillations. Isolated neurons should oscillate, albeit out of phase with each other, unless the autocrine feedback loop is disrupted. Other modelling work has shown similar results concerning the possibility of the neurotransmitter helping to drive neuronal oscillations [17-19], and recent experimental data also offer support for this finding: in mice with genetic lesions of the VPAC2 receptor oscillations of individual neurons are compromised [32]. A subset of neurons is unable to oscillate, whilst other neurons remain rhythmic, although quite desynchronized from each other. In future experimental work it would be interesting to see whether knocking out other candidate coupling mechanisms, such as Prokineticin 2 [33], in tandem could increase the number of neurons not capable of self-sustained oscillations.

We investigated further the properties of the top fifty parameter sets found through our optimization. First we examined the oscillatory state of the solutions in order to see whether the oscillations were driven by the neurotransmitter, as in the optimal parameter set. (This was carried out by calculating the eigenvalues of the Jacobian, evaluated at the unique fixed point given by the single-cell core oscillator, see Additional file 3 for details). When the neurotransmitter loop was included (it is conceivable that there is an autocrine feedback of the neurotransmitter released by a given cell), all parameter sets exhibited an unstable fixpoint and sustained oscillations. However, without the neurotransmitter loop but with a fixed neurotransmitter mean field level, corresponding to the average level for the four-dimensional single-cell core oscillator, 44 parameter sets showed a stable fixed point with imaginary eigenvalues, corresponding to damped (not self-sustained) oscillations (See Additional file 3: Supplementary Table S1). It thus appears that damped single-cell oscillators could be advantageous for efficient synchronization. It would be interesting to conceive experimental strategies to test this prediction. Further, the present study suggests quite general qualitative properties of coupled damped oscillators, and sets the stage for a more rigorous analytical approach to our numerical findings.

We then went on to examine the distributions of the optimized parameters and their correlations, in order to gain some insight into the design of the 50 networks. The parameters of these are quite variable (Additional file 1: Supplementary Figure S2). Of particular note is the fact that synchronised oscillations were possible for Hill coefficients varying from 0.7 – 9.8. Previous models have required high Hill coefficients, representing highly cooperative binding of proteins, in order to generate oscillations, although there is no experimental evidence for this. In fact, for a single three-variable genetic circuit of Goodwin-type with linear decay terms [34], the Hill coefficient must be greater than 8 in order to generate self-sustained oscillations [35].

We found no significant correlations between a single variable and the CF, which is not unexpected due to the nonlinearity and complexity of the system. However, two weak correlations deserve mentioning: First, a low value of the Michaelis constant for the mRNA decay seems to be slightly advantageous for effective synchronization, (*K*_2 _and the CF had a correlation coefficient of 0.16). Second, a low *V*_max _for *Y *protein degradation also seems to be slightly favourable for the synchronization (*v*_4_) and the CF had a correlation coefficient of 0.19). Correlations were stronger between the parameters themselves (Additional file 3: Supplementary Table S2), most notably between the Hill coefficient and *v*_6_, the *V*_max _value for *Z *degradation (correlation coefficient -0.52). In other words, a capability for a high degradation of the transcriptional inhibitor relaxes the demand for a switch-like transcriptional inhibition of *X*. Furthermore, although it seems that it is an advantage for effective synchronization if the single cell core oscillator exhibits damped oscillations (with a constant mean-field level), it seems not to matter *how *damped it is. More technically, this is because the real part of the largest eigenvalue of the system, using the fixed average mean field, was uncorrelated to the cost function (correlation coefficient 0.05), which suggests that the absolute value of the real part of this eigenvalue is not important for a robust multicellular oscillator, as long as it is negative. Last, we calculated the half-lives for the clock mRNA *X*, clock protein *Y*, and transcriptional inhibitor Z, respectively (see Additional file 3: Supplementary Table S3). We found a significant positive correlation between the mRNA half-life and the value of the cost function (see Additional file 1: Supplementary Figure S3). Thus, a prediction from our model is that a not too long half-life for a clock mRNA molecule, perhaps less than an hour, makes efficient synchronization easier.

### Fast re-entrainment is possible with optimal parameter set

**Figure 3:**
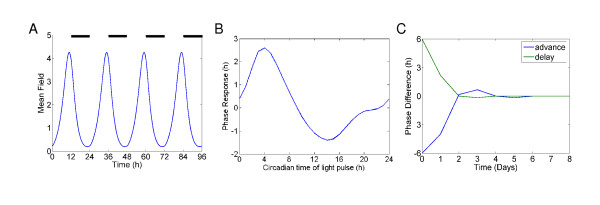
**Phase resetting properties of neurotransmitter driven coupled oscillators**. A. Mean field of neurotransmitter for 200 coupled circadian oscillators entrained by a 12:12 light-dark cycle. The LD cycle is described by a square-wave forcing: *L *= 0 in dark phases and *L *= 1 in light phases. B. Phase response curve for 200 neurotransmitter coupled oscillators. Phase shifts in mean field generated by 1 h light treatments of intensity 1.0 are plotted against the circadian time at which the light pulses were given. Phase advances are plotted as positive values, and delays are plotted as negative values. C. 'Jet lag' experiment for 200 neurotransmitter coupled oscillators. Light dark cycle is advanced or delayed by 6 hours, and then phase difference is calculated by comparison to unperturbed system's phase in LD cycle. Light intensity in LD cycles = 1.0

We went on to investigate the entrainment properties of our optimal parameter set, to see whether we were able to simulate the fast re-entrainment of the SCN seen experimentally. Figure 3A shows that the mean field of the neurotransmitter is stably entrained in a 12:12 LD cycle with light intensity of 1.0, showing no signs of quasi-periodicity as previously reported [17]. We then tested the response of our network with the optimal parameter set to 'jet lag' type experiments, where the LD cycle is advanced or delayed by 6 h, and the resulting phase difference to an unperturbed system is measured. Excitingly, using a light intensity of 1 for the LD cycles which gives only a maximal phase shift of 2.5 h if applied for 1 h (Figure 3B), the mean field oscillations show very fast re-entrainment similar to that seen experimentally: After 1–2 days, for both a 6 h advance and delay, (Figure 3C), there is virtually no difference in phase between the perturbed and unperturbed systems, as seen in experiment [20,21]. We see a slight 'overshoot' effect after a 6 h advance in the light cycle, where the advance in phase of the clock becomes larger than 6 h, then retards to the appropriate steady-state advance over the following days, which is qualitatively similar to the overshoot seen experimentally [21]. The overshoot size was dependent on the light level used in our simulation. Using a lower light level, that results in a maximum phase shift of 0.5 hours if applied for one hour, the system takes around 6 days to re-entrain (Additional file 1: Supplementary Figure S4), with no overshoot effect seen. The actual SCN shows a more pronounced and prolonged desynchronization after phase advances than delays compared to our simulations [21,22], and also shows heterogeneity in the phase resetting properties of individual neurons [36]. It is not possible to simulate this complexity using the simplified SCN model we study. However, these results do show that the experimentally seen extremely fast phase resetting of the SCN after a shift in LD cycle is possible when the neuropeptide positive feedback loop drives the oscillation.

### Light activated pacemaker cells can synchronize the SCN

**Figure 4:**
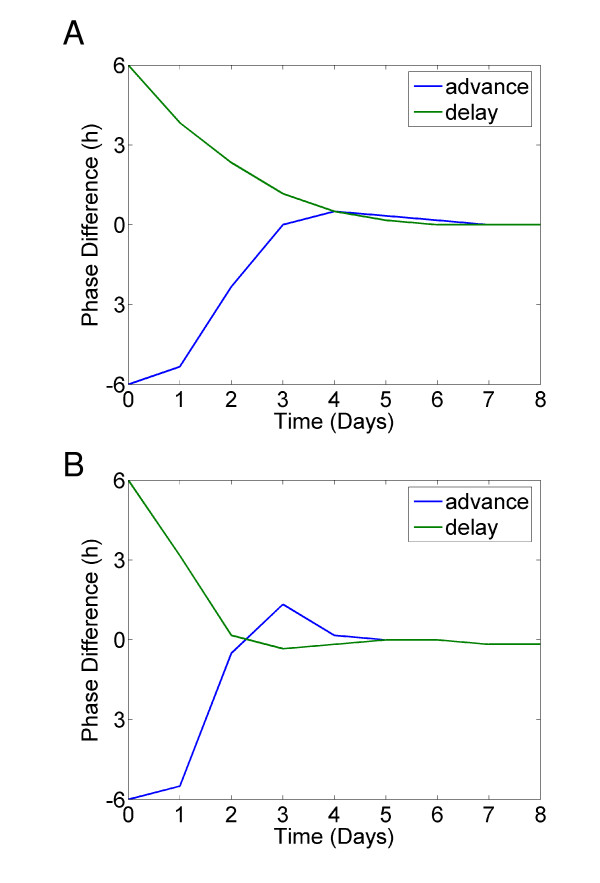
**Light activated oscillators can drive the SCN**. A. 'Jet lag' experiment for 200 neurotransmitter coupled oscillators. 25% of oscillators are light receptive, light intensity = 1.0. B. 'Jet lag' experiment for 200 neurotransmitter coupled oscillators. 25% of oscillators are light receptive, light intensity = 2.0

It has been shown that only a subset of the SCN neurons receives direct light input from the retina. We tested whether the results we observe still hold true under the more physiological assumption that only a subset of the simulated neurons are light activated [37]. Figure 4A shows the results of a 'jet lag' type experiment where only 25% of the cells are light activated with a light intensity of 1.0. Compared to a simulation where all the neurons are light activated (Figure 3), re-entrainment takes longer, about 4 days. However, if we double the strength of the light activation into the SCN, (Figure 4B), we regain fast re-entrainment of 1–2 days. This fits with experimental data showing that PER1 is strongly light activated in light responsive cells [37,38].

## Conclusion

We have used a global parameter scan of a previously developed simplified model of the mammalian circadian clock (Figure 1) [17] to reveal several important design principles. First, we have shown that synchronisation is possible and perhaps more efficient if the individual oscillators are damped (i.e. are not self-sustained) without an oscillatory neuropeptide concentration (Figure 2) – 88% of the best parameter combinations corresponded to damped individual oscillators. This is supported by recent experimental results [32], which showed that without efficient VIP/VPAC2 signalling, a subset of cells are unable to oscillate in a self-sustained manner. We argue that this result, showing that damped core oscillators are preferred for efficient synchronization, has particular weight, since it in no way is due to model assumptions, but rather emerges from the evaluation of a million randomly selected parameter combinations. Second, we have observed that the experimentally seen extremely fast phase resetting of the SCN after a shift in LD cycle can occur in our model when the neuropeptide positive feedback loop drives the oscillation (Figure 3). Future work should test whether fast resetting of the SCN really requires the neuropeptide feedback loop to drive the oscillation. Finally, we see that fast resetting is still possible under the more physiological assumption that only a subset of the simulated neurons are light activated (Figure 4).

Several mathematical models have been constructed that have revealed important aspects of the function of the feedback loops of the mammalian circadian clock [5-7,39]. Recently modelling has also been used to directly understand as well as propose experiments [40,41]. Our work shows that unexpected behaviours can be revealed through a thorough scan of parameter space, even with an extremely simplified model of the mammalian clock. The fact that fast phase resetting of the mammalian clock appears possible when the individual oscillators are not self-sustained has profound implications for our understanding of the experimentally observed result that sub-populations of 'uncoupled' neurons have been seen to be arrhythmic [9]. Simulations of the coupled system of damped oscillators showed robust oscillations even with large variations in the periods of the individual oscillators (Figure 2D), which fits with recent experimental evidence that coupling of the SCN neurons can make the system more robust to genetic perturbations [10]. It also appears that having weakly rhythmic individual oscillators that are strongly coupled can make the system more responsive to external cues such as light (Figure 3).

The SCN structure is extremely complex [42]. There is a great deal of spatial heterogeneity, and individual neurons have been shown to differ in their neuropeptide expression, light responsiveness, phase [43], and free running period. This complexity cannot be reproduced with the extremely simplified model of the SCN used in our study. In future it would be interesting to study the heterogeneity of the SCN, and to examine a more realistic model of its function. However, as we have seen, simplified models are extremely useful for uncovering general mechanisms of genetic networks. More detailed models of the SCN require an increase in the number of unknown parameters [18,19], and this makes our global parameter search method less comprehensive.

We found the optimized parameters for the core negative feedback loop to be extremely varied (Additional file 1: Supplementary Figure S2). A single-cell model with a Hill coefficient of repression as low as 0.7 was still able to function as the building block of a robustly oscillating cellular network when driven by the neuropeptide mean-field. This is very different behaviour from previous circadian clock models, where a high value of the Hill coefficient was crucial to obtain limit cycle oscillations [35]. Our work shows that future experiments and mathematical models must take into account the neuropeptide feedback loop in order to understand the function of the mammalian clock, even at the single neuron level.

## Authors' contributions

JCWL, PW and HH conceived and designed the study. JCWL and PW performed the numerical experiments. JCWL, PW, HH, and AK analyzed the data. All authors contributed to writing the paper, and read and approved the final manuscript.

## Supplementary Material

Additional file 1 Supplementary figures 1–4. Click here for file

Additional file 2 Supplementary figure legends. Click here for file

Additional file 3 Supplementary information and supplementary tables. Click here for file
