# Supplementary figures and images for: Global parameter search reveals design principles of the mammalian circadian clock
Source: BMC Syst Biol. 2008 Feb 29;2:22. doi: 10.1186/1752-0509-2-22 (PMC2277373; doi:10.1186/1752-0509-2-22)

Sup figure 1

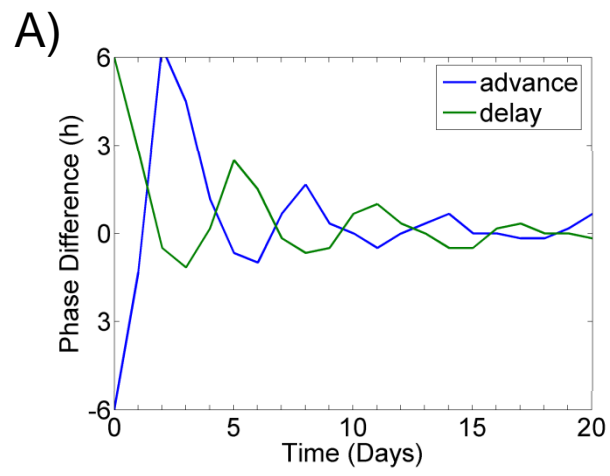

Sup figure 2

A)

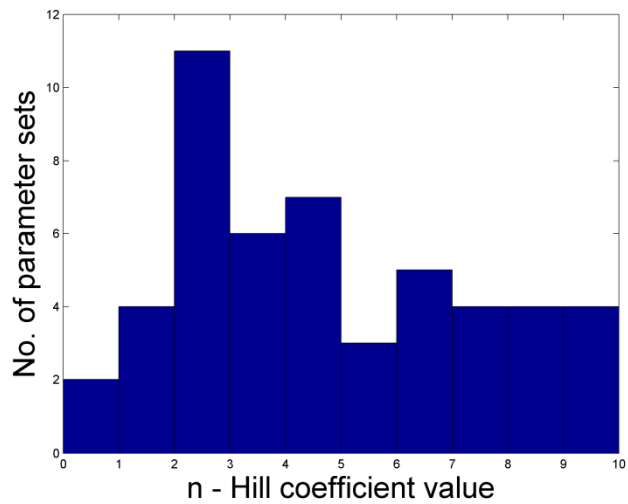

B)

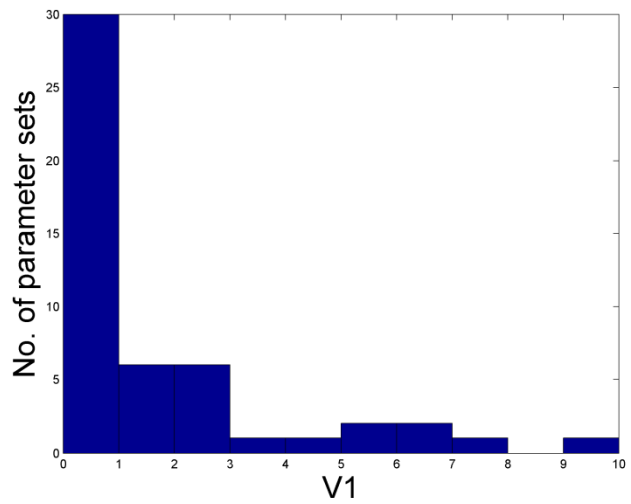

C)

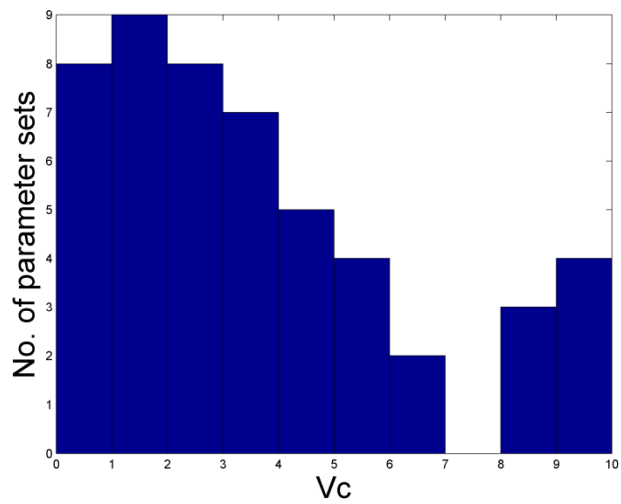

D)

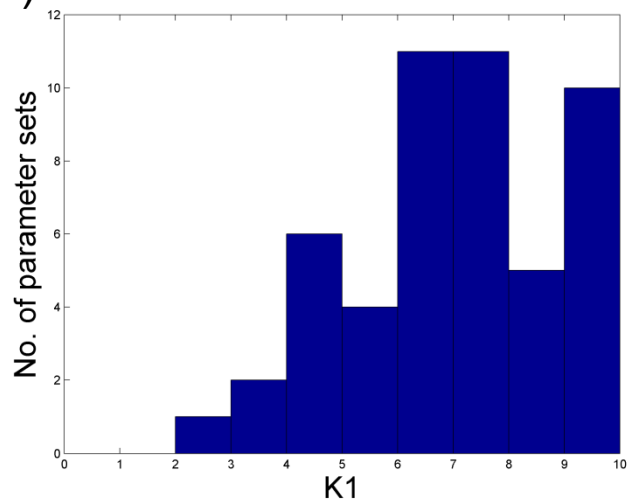

Sup figure 3

A)

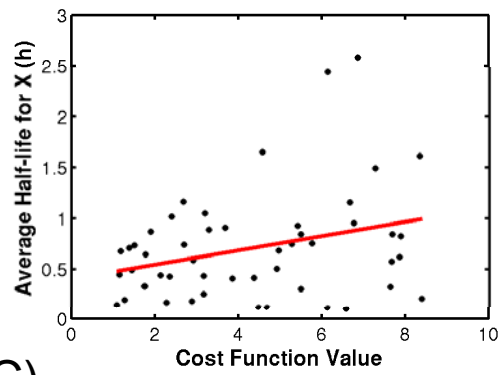

B)

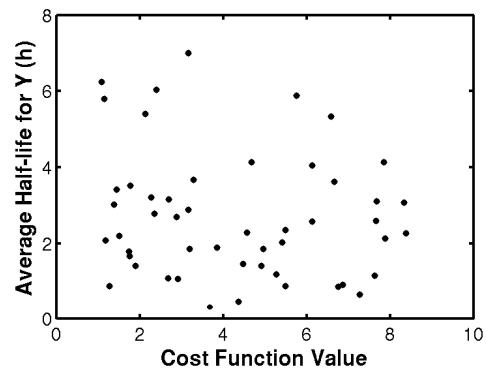

C)

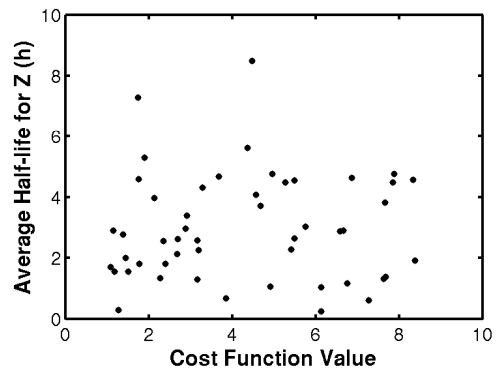

Sup figure 4

A)

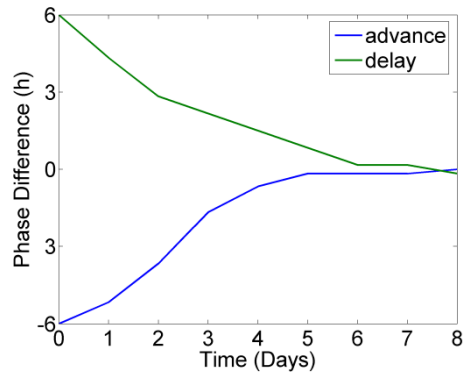

B)

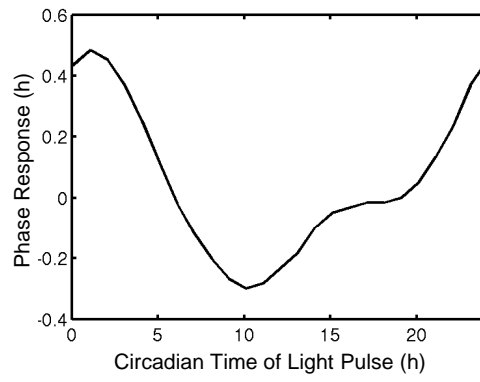

Supplement: Additional file 1 — Supplementary figures 1–4. [file 1752-0509-2-22-S1.pdf]
