# Supplementary material for: Global parameter search reveals design principles of the mammalian circadian clock
Source: BMC Syst Biol. 2008 Feb 29;2:22. doi: 10.1186/1752-0509-2-22 (PMC2277373; doi:10.1186/1752-0509-2-22)
Supplement: Additional file 2 — Supplementary figure legends. [file 1752-0509-2-22-S2.pdf]

Supplementary Figure Legends

Supplementary Figure 1: Phase resetting properties of 200 coupled oscillators using parameter values taken from Gonze et al. (2005).
"Jet lag" experiment for 200 neurotransmitter coupled oscillators. Light-dark cycle is advanced or delayed by 6 hours, and then phase difference is calculated by comparison to unperturbed system. Light intensity in LD cycles = 0.01.

Supplementary Figure 2: Analysis of distribution of top 50 parameter values.

a) Distribution of n, the Hill coefficient, for top 50 optimized parameter values
b) Distribution of V1, transcription rate of X, for top 50 optimized parameter values
c) Distribution of Vc, for top 50 optimized parameter values
d) Distribution of K1, the Michaelis Menten coefficient for X transcription, for top 50 optimized parameter values

Supplementary Figure 3: Comparison of average half life of core clock components and Cost Function score for the top 50 parameters sets.

3a) Average half life of *X* versus CF score

3b) Average half life of *Y* versus CF score

3c) Average half life of *Z* versus CF score

Supplementary Figure 4: Analysis of phase resetting properties of coupled oscillators at low light levels

4a) "Jet lag" experiment for 200 neurotransmitter coupled oscillators at low light levels. Light dark cycle is advanced or delayed by 6 hours, then phase difference is calculated by comparison to unperturbed system. Light intensity in LD cycles = 0.2.

4b) Phase response curve for 200 neurotransmitter coupled oscillators at low light levels. Phase shifts in mean field generated by 1 h light treatments of

intensity 0.2 are plotted against the circadian time at which the light pulses were given. Phase advances are plotted as positive values, and delays are plotted as negative values.
