# Supplementary material for: Global parameter search reveals design principles of the mammalian circadian clock
Source: BMC Syst Biol. 2008 Feb 29;2:22. doi: 10.1186/1752-0509-2-22 (PMC2277373; doi:10.1186/1752-0509-2-22)
Supplement: Additional file 3 — Supplementary information and supplementary tables. [file 1752-0509-2-22-S3.pdf]

Supplementary Information

Computational Model

To simulate circadian oscillations in coupled mammalian cells, we use a simplified model based on the Goodwin oscillator [Goodwin, 1965], as described in the paper by Gonze et al. [2005]. For full details of the assumptions and reasoning behind this model, please see Gonze et al. [2005], but we summarise the main points here. In this model, a clock gene mRNA (X) produces a clock protein (Y) which, in turn, activates a transcriptional inhibitor (Z). A negative feedback loop is then formed by Z repressing the transcription of X mRNA. A Hill function is used to describe the transcriptional activation term for X, and Michealis-Menten kinetics are used to describe enzyme mediated degradation.

We took the following as our mathematical model for a system of N oscillators (denoted by ($i = 1, 2, \ldots, N$):

$$\frac{dX_i}{dt} = v_1 \frac{K_1^n}{K_1^n + Z_i^n} - v_2 \frac{X_i}{K_2 + X_i} + v_c \frac{KF}{K_c + KF} + L, \tag{1}$$

$$\frac{dY_i}{dt} = k_3 X_i - v_4 \frac{Y_i}{K_4 + Y_i}, \tag{2}$$

$$\frac{dZ_i}{dt} = k_5 Y_i - v_6 \frac{Z_i}{K_6 + Z_i}. \tag{3}$$

As well as through the negative feedback loop, X levels are modulated by light (L) and intercellular coupling through the mean field F of the neurotransmitter. Light is assumed to act independently of the negative feedback loop through the additive term L(t) (In dark conditions L = 0). We assume that cells synthesize a neuropeptide (V), whose production is induced by the activation of X.

$$\frac{dV_i}{dt} = k_7 X_i - v_8 \frac{V_i}{K_8 + V_i} \tag{4}$$

The neurotransmitter release is taken to be fast compared to the 24 h timescale of the oscillations. The neurotransmitter levels thus become homogeneous, which results in an average neurotransmitter level, or a mean field F:

$$F = \frac{1}{N}\sum_{i=1}^{N} V_i. \tag{5}$$

The coupling strength parameter (K) in Eq. 1 describes how sensitive the individual oscillator is to the neurotransmitter levels. We initially set K to 1.0, leaving 16 parameters to determine using our parameter optimization scheme.

Parameter Optimisation

In order to optimise the parameters for the study, we followed the optimisation scheme as developed in the papers by Locke et al. [2005a,b]. The equations were solved using Matlab, integrated using the equation solver ode45. The optimisation process described in the following sections was carried out by compiling the Matlab code into C and running the code on a task farm super computer consisting of 31 x 2.6 GHz Pentium4 Xeon 2-way SMP nodes (62 CPUs in total). In order to evaluate the terms of the cost function, we solved numerically Eqns 1-5 over 1000 h in constant darkness (DD) (the first 600 h of each solution are discarded as transitory). We initialised our simulation by setting all concentrations initially to 1.

First we constructed a cost function Δ, which consists of error terms that check that the individual oscillators are synchronised, and that the mean field oscillates on a limit cycle with a period of approx 24 h under constant dark conditions.

The cost function is defined as:

$$\Delta = 1/R + \delta_{\tau_{\mathrm{d}}} + \delta_{\phi} + \delta_{\mathrm{size}} \tag{6}$$

we now describe each term of the cost function, equation 6, in turn.

To quantify how good the synchrony is, we calculate an order parameter R [Gonze et al., 2005]:

$$R = \frac{\langle F^2 \rangle - \langle F \rangle^2}{\frac{1}{N}\sum_{i=1}^{N}\left(\langle V_i^2 \rangle - \langle V_i \rangle^2\right)} = \frac{\mathrm{Var}_t(F)}{\mathrm{Mean}_i(\mathrm{Var}_t(V_i))}, \tag{7}$$

where $\langle \cdot \rangle$ denotes the average over time. This parameter measures the distribution of phases of the oscillators and is ranging between 0 (no synchronization) and 1 (perfect synchronization, with all oscillators in phase). Our first Δ term is 1/R, giving low error scores for well synchronised solutions.

Next, $\delta_{\tau_{\mathrm{d}}}$ measures the difference between the experimental target period and the mean period of the oscillation of the mean field F in constant darkness (DD).

$$\delta_{\tau_{\mathrm{d}}} = \frac{1}{M}\sum_{j=1}^{M}(24 - \tau_F^{(j)})^2 \tag{8}$$

This is the error in the period, τ_F, of the mean field oscillations under DD, averaged over the M cycles between $600 < t < 900$. A marginally acceptable period difference of 1h contributes O(1) to the cost function.

Secondly, δ_ϕ ensures that the mean field oscillation is on a limit cycle,

$$\delta_\phi = \left(\frac{\sigma[F(t_{\mathrm{p}})]_{\mathrm{d}}}{0.01\langle F(t_{\mathrm{p}})\rangle_{\mathrm{d}}}\right)^2 + \left(\frac{F({t_{\mathrm{p}}}^M) - F({t_{\mathrm{p}}}^1)}{0.05F({t_{\mathrm{p}}}^M)}\right)^2 + \left(\frac{\Delta F(M) - \Delta F(1)}{0.05\Delta F(M)}\right)^2 \tag{9}$$

The first term ascribe a cost of O(1) for limit cycle solutions in DD cycles whose peak heights, $F(t_{\mathrm{p}})$, vary only within 1 percent of one another. Here, $\langle\rangle_{\mathrm{d}}$ is the average over the cycles in DD between $600\,\mathrm{h} < t < 900\,\mathrm{h}$, and $\sigma[]_{\mathrm{d}}$ is the standard deviation for the cycles in DD between $600\,\mathrm{h} < t < 900\,\mathrm{h}$. The next two terms were added to check that the oscillation amplitude ($\Delta F = F_{\mathrm{max}} - F_{\mathrm{min}}$), and peak heights of the mean field oscillations, were not steadily increasing or decreasing in size from the first (1) to last cycle (M) examined.

Finally δ_{size} checks that the oscillation amplitudes are not too small

$$\delta_{\mathrm{size}} = \left(\frac{0.01}{\langle \Delta F \rangle_{\mathrm{d}}}\right)^2 \tag{10}$$

The term introduces a > 1 cost for solutions in DD cycles with oscillation amplitude, ΔF, less than 0.01.

We implemented the Antoneev-Saleev variant of the Sobol quasi-random number generator to choose parameter values in our sixteen-dimensional parameter space, adapted from Press et al. [1996]. The initial values as described in the paper by Joe and Kuo [2003] were used, allowing number generation in up to 1111 dimensions. We bounded the parameter space for the 16 parameters to be optimised to be bounded $\in [0, 10]$, where our typical

parameter scale is unity in nM=hours=1. We calculated Δ a set of 20 coupled oscillators for one million Sobol points, and the top 150 solutions found from this search of parameter space were then passed onto a further screen. These 150 parameter sets were solved for a set of 100 coupled oscillators, and the cost function was solved for ten different initial conditions of periods, and averaged. The top 50 paramater sets from these 150 were then used as our reference solutions.

Calculation of the Stability of Fixed Points and Limit Cycles

In order to assess the dynamics of single non-coupled oscillators, we performed linear stability analysis of the fixed points, as well as checking for stable periodic solutions via numerical time integration.

The core oscillator, three-dimensional with a fixed neurotransmitter variable V and hence a fixed mean field F, either has one unique fixed point, or no fixed point at all. This may be realized from the following reasoning. In order to obtain $dY/dt = 0$, we get $Y = k_3K_4X/\left(v_4 - k_3X\right)$, a monotonically increasing function of X as long as we consider only physical non-negative solutions, i.e. $v_4 - k_3X > 0$. In the same way, from the equation for $dZ/dt = 0$, we see that Z is a monotonically increasing function of Y, and therefore also of X. In order to obtain $dX/dt = 0$, we get

$$\frac{v_1 K_1^n}{K_1^n + Z\left(X\right)^n} + \frac{v_c K F}{K_c + KF} = \frac{v_2 X}{K_2 + X}. \tag{11}$$

Since the lefthand term is monotonically decreasing towards $v_cKF/\left(K_c + KF\right)$ and the righthand term is monotonically increasing from zero as X increases, this equation has exactly one solution for $X > 0$ as long as $v_2 > v_cKF/\left(K_c + KF\right)$, zero solutions otherwise.

For the four-dimensional model including the autocrine feedback loop, we get the additional equation $dV/dt = 0$ for the fixed point. This gives $V = k_7K_8X/\left(v_8 - k_7X\right)$, which monotonically increases with increasing X. The mean field $F\left(X\right)$ for the four-dimensional single-cell oscillator is equal to V. This means that the left-hand side is no longer monotonic with respect to X, which in turn means that there is a possibility of having two fixed points. We analyzed equation 11 for all the 50 best parameter sets, and found for each set one unique fixpoint, both with fixed and variable mean field.

We applied linear stability analysis by analyzing the eigenvalues of the Jacobian of equation system 1–5 for a single-cell oscillator at the unique

fixpoint for each of the top 50 parameter sets. For a fixed mean field, the Jacobian of the resulting three-dimensional system is:

$$J_{\text{fix}} = \begin{pmatrix} -\frac{v_2 K_2}{(K_2+X)^2} & 0 & -\frac{v_1 K_1^n n Z^{n-1}}{\left(K_1^n+Z^n\right)^2} \\ k_3 & -\frac{v_4 K_4}{(K_4+Y)^2} & 0 \\ 0 & k_5 & -\frac{v_6 K_6}{(K_6+Z)^2} \end{pmatrix},$$

while for the four-dimensional oscillator with autocrine feedback from the neurotransmitter variable V, the Jacobian is written

$$J_{\text{full}} = \begin{pmatrix} -\frac{v_2 K_2}{(K_2+X)^2} & 0 & -\frac{v_1 K_1^n n Z^{n-1}}{\left(K_1^n+Z^n\right)^2} & \frac{v_c K_c}{(K_c+V)^2} \\ k_3 & -\frac{v_4 K_4}{(K_4+Y)^2} & 0 & 0 \\ 0 & k_5 & -\frac{v_6 K_6}{(K_6+Z)^2} & 0 \\ k_7 & 0 & 0 & -\frac{v_8 K_8}{(K_8+V)^2} \end{pmatrix}.$$

Eigenvalues for these Jacobians were calculated numerically. Along with each set of eigenvalues, we also integrated the equation system 1–5. In all cases, we observed a stable limit cycle when the fixed point was unstable, i.e. when at least one eigenvalue had a positive real part, and damped oscillations when the fixed point was stable. The results are summarized in table 1.

Calculation of Half-lives

We chose our definition of half-lives to be the same as the one used in most experimental settings. Thus, given an initial condition, imagine that the production of a species, say X, is abruptly arrested. The equation for decay is now

$$\frac{dX}{dt} = -\frac{vX}{K+X},$$

assuming Michaelian degradation, as in the full model. Separating the variables and integrating, we obtain

$$K \log X + X = -vt + C,$$

where C is an integration constant. This constant may be evaluated at the initial concentration $X(0)$ at $t = 0$, which gives $C = K \log X(0) + X(0)$. We then obtain

$$\frac{K}{v} \log \frac{X(0)}{X} + \frac{1}{v}\left(X(0) - X\right) = t.$$

We now search the half-life, at which the X is half of its initial value. Inserting $X = X(0)/2$ yields the half-life $t_{1/2}$:

$$t_{1/2} = \frac{K}{v} \log 2 + \frac{1}{2v} X(0).$$

For each parameter set, half-lives for X, Y, and Z were calculated. This was done for the full four-dimensional system with the autocrine feedback loop, for one full period, i.e. one lap around the limit cycle. Half-lives were then averaged. Results are presented in supplementary figure 3. Of particular note is that the half-live for cytosolic protein (Y) generally is in the order of hours, which agrees with recent experimental data for PER2 [Vanselow et al., 2006]. Correlation coefficients between the half-lives and the cost function are given in table 3.

Tables

Table 1: Results for the analysis of the stability of limit cycle and fixed points. Shown is the number of parameter sets from the top fifty optimized parameter sets that showed either unstable, damped or overdamped behaviour for the 4 dimensional model including the autocrine feedback loop and the three dimensional model without. The three-dimensional system had a fixed mean-field corresponding to the mean of the mean-field of the corresponding four-dimensional system.

	Three-dimensional	Four-dimensional
Unstable (spiral repellor)	6	50
Damped (spiral node)	43	
Overdamped (node)	1	

Table 2: Significant correlations between parameters ($p < 0.05$).

Parameter 1	Parameter 2	Correlation Coefficient
v_c	k_7	-0.42
k_7	v_8	0.28
k_7	K_8	-0.28
v_8	K_8	0.37
v_8	n	0.29
v_1	v_2	0.31
v_1	v_4	0.29
n	v_4	-0.29
k_3	v_4	0.39
v_c	K_4	-0.29
v_4	k_5	-0.51
v_c	v_6	0.28
k_7	v_6	-0.36
n	v_6	-0.52

Table 3: Correlations between the calculated half-lives and the cost function. (**) $p < 0.05$.

Variable	Correlation Coefficient
X	0.30 (**)
Y	-0.16
Z	0.038

References

B. C. Goodwin. Oscillatory behavior in enzymatic control processes. *Adv. Enzyme Regul*, 3:425–438, 1965.

D. Gonze, S. Bernard, C. Waltermann, A. Kramer, and H. Herzel. Spontaneous synchronization of coupled circadian oscillators. *Biophys J.*, 89: 120–129, 2005.

J. C. W. Locke, A. J. Millar, and M. S. Turner. Modelling genetic networks with noisy and varied data: The circadian clock in Arabidopsis thaliana. *J. Theor. Biology*, 234:383–393, 2005a.

J. C. W. Locke, M. M. Southern, L. Kozma-Bognar, V. Hibberd, P. E. Brown, M. S. Turner, and A. J. Millar. Extension of a genetic network model by

iterative experimentation and mathematical analysis. *Mol Syst Biol*, 1: 2005.0013, 2005b.

W. H. Press, S. A. Teukolsky, W. T. Vettering, and B. P. Flannery. *Numerical Recipes in C: The Art of Scientific Computing.* Cambridge, Cambridge, 1996.

S. Joe and F. Y. Kuo. Remark on ALGORITHM 659: implementing Sobol's quasirandom sequence generator. *ACM Trans. on Mathematical Software (TOMS) archive*, 29:49–57, 2003.

K. Vanselow, J. T. Vanselow, P. O. Westermark, S. Reischl, B. Maier, T. Koarte, A. Herrmann, H. Herzel, A. Schlosser, and A. Kramer. Differential effects of PER2 phosphorylation: molecular basis for the human familial advanced sleep phase syndrome (FASPS). *Genes Dev*, 20:2660–72, 2006.
